# Supplementary material for: Cerebrospinal fluid concentration of complement component 4A is increased in first episode schizophrenia
Source: Nat Commun. 2022 Nov 3;13:6427. doi: 10.1038/s41467-022-33797-6 (PMC9633609; doi:10.1038/s41467-022-33797-6)
Supplement: Supplementary file 1 — Supplementary Information [file 41467_2022_33797_MOESM1_ESM.pdf]

Supplementary Information for

Cerebrospinal fluid concentration of complement component 4A is increased in first-episode schizophrenia

Author list: Jessica Gracias[1], Funda Orhan[1], Elin Hörbeck[2,3], Jessica Holmén-Larsson[2], Neda Khanlarkani[1], Susmita Malwade[1], Sravan K. Goparaju[1], Lilly Schwieler[1], İlknur Ş. Demirel[1], Ting Fu[4], Helena Fatourus-Bergman[5,6], Aurimantas Pelanis[7], Carleton P. Goold[8], Anneli Goulding[3,9], Kristina Annerbrink[3], Anniella Isgren[2,3], Timea Sparding[2], Martin Schalling[10], Viviana A. Carcamo Yañez[11], Jens C. Göpfert[11], Johanna Nilsson[2], Ann Brinkmalm[2], Kaj Blennow[2,12], Henrik Zetterberg[2,12,13,14,15], Göran Engberg[1], Fredrik Piehl[5], Steven D. Sheridan[4], Roy H. Perlis[4], Simon Cervenka[5,6,16], Sophie Erhardt[1], Mikael Landen[2,17], Carl M. Sellgren[1,6*]

Affiliations: [1]Department of Physiology and Pharmacology, Karolinska Institutet, Stockholm, Sweden; [2]The Institute of Neuroscience and Physiology, University of Gothenburg, Gothenburg, Sweden; [3]Psychosis Clinic, Sahlgrenska University Hospital, Mölndal, Sweden; [4]Center for Quantitative Health, Center for Genomic Medicine and Department of Psychiatry, Massachusetts General Hospital, Boston, MA, USA; [5]Department of Clinical Neuroscience, Karolinska Institutet, Stockholm, Sweden; [6]Stockholm Health Care Services, Region Stockholm, Sweden; [7]Department of Anesthesiology, Sahlgrenska University Hospital, Gothenburg, Sweden; [8]Novartis Institutes of BioMedical Research, Cambridge, MA, USA; [9]Department of Psychology, University of Gothenburg, Gothenburg, Sweden; [10]Department of Molecular Medicine and Surgery, Karolinska Institutet and Center for Molecular Medicine, Karolinska University Hospital, Stockholm, Sweden; [11]NMI Natural and Medical Sciences Institute at the University of Tübingen, Reutlingen, Germany; [12]Clinical

Neurochemistry Laboratory, Sahlgrenska University Hospital, Mölndal, Sweden; [13]Department of Neurodegenerative Disease, UCL Institute of Neurology, Queen Square, London, UK; [14]UK Dementia Research Institute at UCL, London, UK; [15]Hong Kong Center for Neurodegenerative Diseases, Hong Kong, China; [16]Department of Medical Sciences, Psychiatry, Uppsala University, Uppsala, Sweden; [17]Department of Medical Epidemiology and Biostatistics, Karolinska Institutet, Stockholm, Sweden.

Table of contents

Supplementary Figure 1. Cerebrospinal fluid levels of C4A in the combined discovery and replication sample.

Supplementary Figure 2. Cerebrospinal fluid levels of C4A after stratifying for the use of antipsychotic medication in the discovery (KaSP) and replication (GRIP) cohort.

Supplementary Figure 3. Cerebrospinal fluid C4A and C4B protein concentration correlated to corresponding Copy numbers (CNs) in the GRIP cohort.

Supplementary Figure 4. Cerebrospinal fluid C4A and C4B protein concentration adjusted for corresponding Copy numbers (CNs) and predicted RNA expression in the GRIP cohort.

Supplementary Figure 5. Comparison of IL-1beta and C4A levels in CSF from patients with FEP.

Supplementary Figure 6. Total C4 concentrations in cerebrospinal fluid from patients with first episode psychosis patients and healthy controls (both cohorts).

Supplementary Figure 7. Correlation of total C4 cerebrospinal fluid levels with age.

Supplementary Figure 8. Correlation of C4A and C4B cerebrospinal fluid levels with age.

Supplementary Figure 9. Cerebrospinal fluid levels of C4A and C4B per respective copy numbers in the combined discovery and replication cohort (all subjects).

Supplementary Figure 10. Distribution of *C4AL*, *C4AS*, *C4BL* and *C4BS* copy numbers in the GRIP cohort.

Supplementary Table 1. Demographic and clinical characteristics of the study participants in the replication sample (KaSP).

Supplementary Table 2. Demographic and clinical characteristics of the study participants in the discovery sample (GRIP).

Supplementary table 3. Correlations of CSF C4A concentration on change in EMG recordings during habituation to pulse alone trials, and, at the interstimulus intervals (ISI).

Supplementary Table 4. Inclusion list of the light and heavy peptides with their theoretical masses (m) and charge states (z).

Supplementary Table 5: Post-hoc analyses of the non-selected but detectable cytokines on the 10-plex panel against CSF C4A.

Supplementary Figure 1.

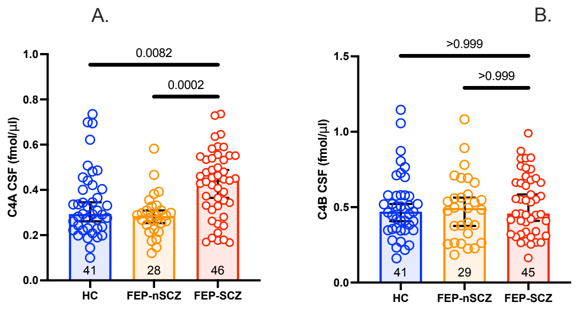

Supplementary Figure 1. Cerebrospinal fluid levels of C4A and C4B in the combined discovery and replication sample. Median age in the combined cohort was 30 years (interquartile range: 25-35), and the cohort consisted of 33 females and 83 males. A. Cerebrospinal fluid (CSF) C4A concentrations were significantly elevated in patients with first-episode psychosis who developed schizophrenia (FEP-SCZ; n=46; Median=0.44 fmol/ul: 95% confidence interval [CI]=0.38-0.47) as compared to healthy controls (HCs; n=41; Median=0.29 fmol/ul: CI=0.29-0.39) or patients with FEP who did not develop schizophrenia (FEP-nSCZ; n=28; Median=0.28 fmol/ul: CI=0.25-0.32). Adjusting the analysis for sex, age, and smoking, C4A levels in patients with FEP-SCZ remained significantly elevated as compared to HCs (mean difference: 0.087; CI=0.028-0.147; p(adjusted)=0.005), and as compared to patients with FEP-nSCZ (mean difference: 0.137; CI=0.071-0.204; p(adjusted)= $8.3x10^{-5}$. B. CSF C4B concentrations were not significantly elevated in patients with FEP-SCZ (n=45; Median=0.46 fmol/ul: CI=0.46-0.58) as compared to HCs (n=41; Median=0.47 fmol/ul: CI=0.44-0.57) or patients with FEP-nSCZ (n=29; Median=0.49 fmol/ul: CI=0.41-0.58). Adjusting the analysis for sex, age, and smoking, C4B levels in patients with FEP-SCZ remained similar as compared to HCs (mean difference: 0.023; CI=-0.063-0.110; p(adjusted)=0.593), and as compared to patients with FEP-nSCZ (mean difference: 0.018; CI=-0.077-0.112; p(adjusted)=0.711).Unadjusted analyses were performed using Kruskal-Wallis *H* test followed by post-hoc tests, and adjusted analyses were performed using ANCOVAs followed by post-hoc-tests. All reported p-values are two-sided. Bar graphs represent medians and error bars represent 95% CIs. Source data for graphs are provided as a Source Data file.

Supplementary Figure 2.

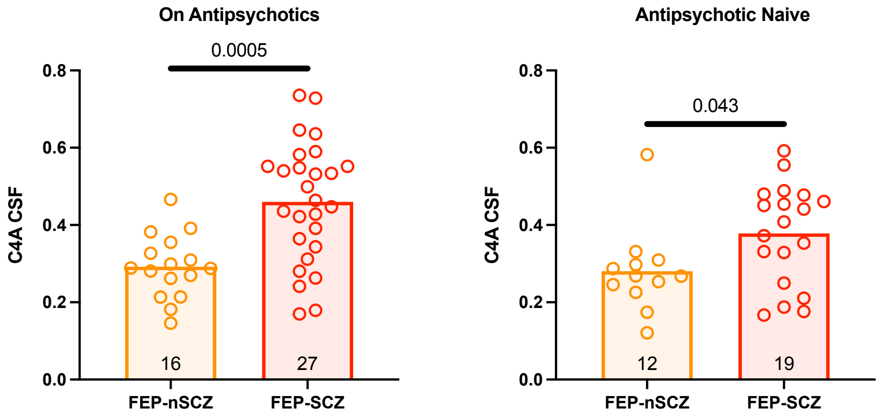

Supplementary Figure 2. Cerebrospinal fluid levels of C4A after stratifying for the use of antipsychotic medication in the discovery (KaSP) and replication (GRIP) cohort. Among the patients in the combined cohort with first-episode psychosis (FEP) who were treated with an antipsychotic agent, the patients who developed schizophrenia displayed higher cerebrospinal fluid (CSF) C4A levels than FEP patients who did not develop SCZ (FEP-SCZ, n=27: median=0.46 fmol/ul: 95% confidence interval [CI]=0.36-0.55, FEP-nSCZ, n=16; median=0.29 fmol/ul: CI=0.21-0.36). Similar, in the antipsychotic naïve group, patients with FEP-SCZ (n=19; median=0.41 fmol/ul: CI=0.25-0.48) displayed higher C4A concentration than patients with FEP-nSCZ (n=12; median=0.27 fmol/ul: CI=0.23-0.31, respectively). Data was analysed using Mann-Whitney *U* tests. The reported p-values are two-sided. Bar graphs represent medians and error bars represent 95% CIs. Source data for graphs are provided as a Source Data file.

Supplementary Figure 3.

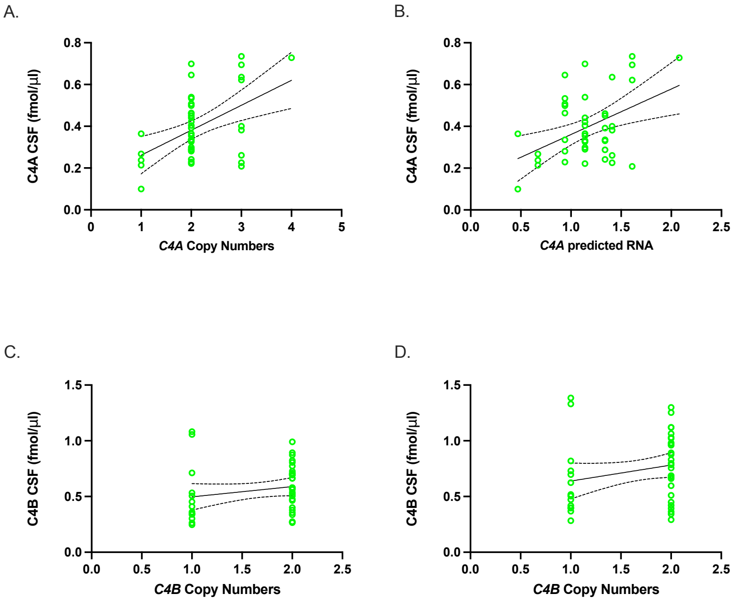

Supplementary Figure 3. Cerebrospinal fluid C4A and C4B protein concentration correlated to corresponding Copy numbers (CNs) in the GRIP cohort. Cerebrospinal fluid (CSF) C4A concentration (fmol/ul) was correlated to *C4A* copy numbers (A. r= 0.48; CI: 0.21-0.68; *P*=0.001) as well as to predicted *C4A* RNA expression (B. *r*=0.44; CI: 0.16-0.65; *P*=0.003). C. CSF C4B protein was not correlated with *C4B* copy numbers *r*=0.19; CI: -0.11-0.46; *P*=0.205), even after re-measuring C4B levels (D. *r*=0.22; CI: -0.08-0.49; *P*=0.146). Data was analysed using a Pearson's correlation (two-sided p-value). Intercept with 95% confidence interval is indicated in the graph. Source data is provided as a Source Data file.

Supplementary Figure 4.

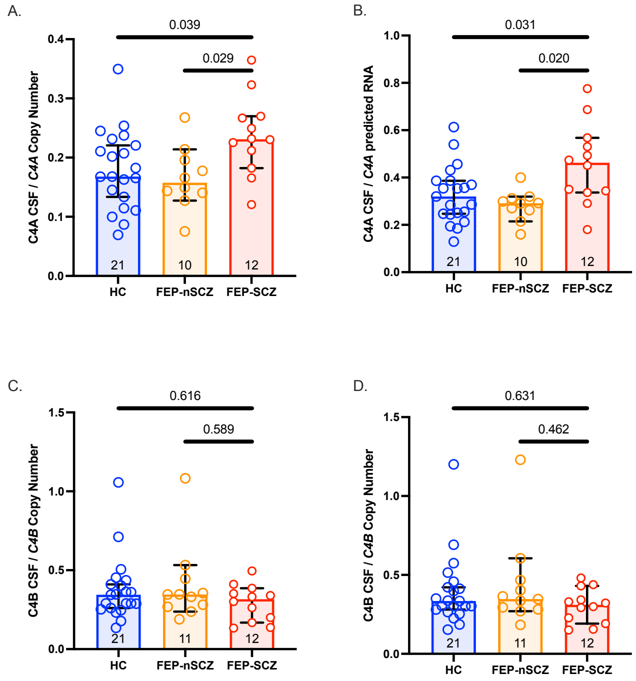

Supplementary Figure 4. Cerebrospinal fluid C4A and C4B protein concentration adjusted for corresponding copy numbers (CNs) and predicted RNA expression in the GRIP cohort. The median age of the cohort was 33 years, and 38 of the 44 participants were males. Cerebrospinal fluid (CSF) C4A concentrations per *C4A* CNs as measured by droplet digital PCR (A.) and per genetically predicted *C4A* predicted RNA expression (B.) were significantly increased in patients with first-episode psychosis who developed schizophrenia (FEP-SCZ: n=12 median=0.23, CI:0.19-0.28 and median=0.46, CI:0.35-0.56; respectively) as compared to healthy controls (HCs: n=21 median=0.17, CI:0.15-0.21 and median=0.32, CI:0.27-0.38; respectively) or patients with FEP who did not develop SCZ (FEP-nSCZ: n=10 median=0.16, CI:0.13-0.20 and median=0.29, CI:0.24-0.33; respectively). However, CSF C4B concentrations per *C4B* CNs (C.) and per genetically predicted *C4B* predicted RNA expression (D.) were similar between FEP-SCZ (n=12 median=0.32, CI:0.22-0.37 and median=0.31, CI:0.24-0.37; respectively) compared to HCs (n=21 median=0.34, CI:0.28-0.47 and median=0.33, CI:0.29-0.50; respectively) and FEP-nSCZ (n= 11 median=0.35, CI: 0.24-0.57 and median=0.35, CI:0.24-0.63; respectively). All data were analysed by Kruskal-Wallis *U* tests and corrected for multiple testing. All reported p-values are two-sided. Bar graphs represent medians and error bars represent 95% confidence interval Source data is provided in the Source Data file.

.

Supplementary Figure 5.

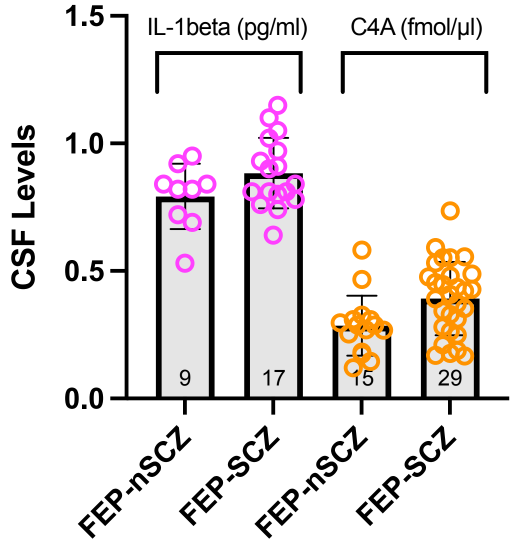

Supplementary Figure 5. Comparison of IL-1beta and C4A levels in CSF from patients with FEP. For comparison reason, IL-1beta and C4A levels in cerebrospinal fluid (CSF) of patients with first episode psychosis who develop schizophrenia (FEP-SCZ) compared to those who do not develop schizophrenia (FEP-nSCZ). CSF C4A levels corresponds to the to last two bar graphs in Figure 1. Bar graphs represent medians and error bars represent 95% confidence interval Source data is provided in the Source Data file.

Supplementary Figure 6.

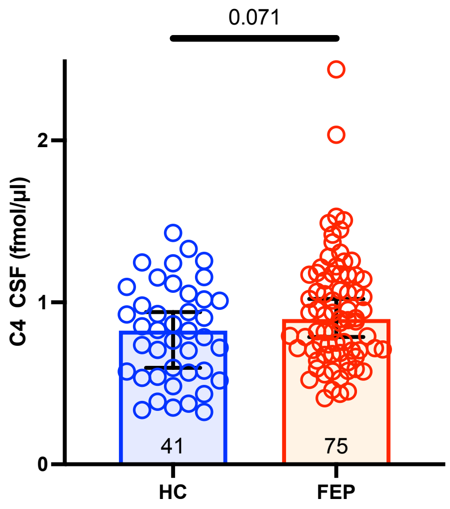

Supplementary Figure 6. Total C4 concentrations in cerebrospinal fluid from patients with first episode psychosis patients and healthy controls (both cohorts). The elevation of total C4 levels in CSF from patients with first-episode psychosis (FEP, n=75) did not reach significance as compared to healthy controls (HCs, n=41). FEP group: median=0.90 fmol/ul, CI:0.86-1.0., HCs: median=0.83fmol/ul, CI=0.71-0.90. Data was analysed using a Mann-Whitney *U* test. The reported p-value is two-sided. Bar graphs represent medians and error bars represent 95% CIs. Source data is provided in the Source Data file.

Supplementary Figure 7.

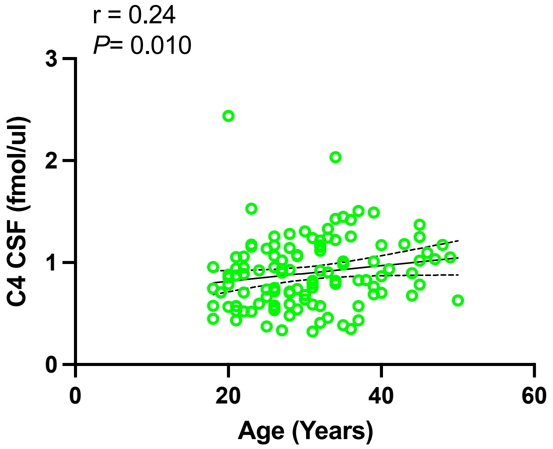

Supplementary Figure 7. Correlation of total C4 cerebrospinal fluid levels with age. Total C4 levels was significantly correlated with age in 116 cerebrospinal fluid samples from both cohorts (median age: 30 years; interquartile range 25-35, 83 males and 33 females) using a Spearman correlation analysis (*r*= 0.24; 95% confidence interval:0.05-0.41; *P*=0.010). Intercept with 95% confidence interval is indicated in the graph, p-value is two-sided. Source data is provided in the Source Data file.

Supplementary Figure 8.

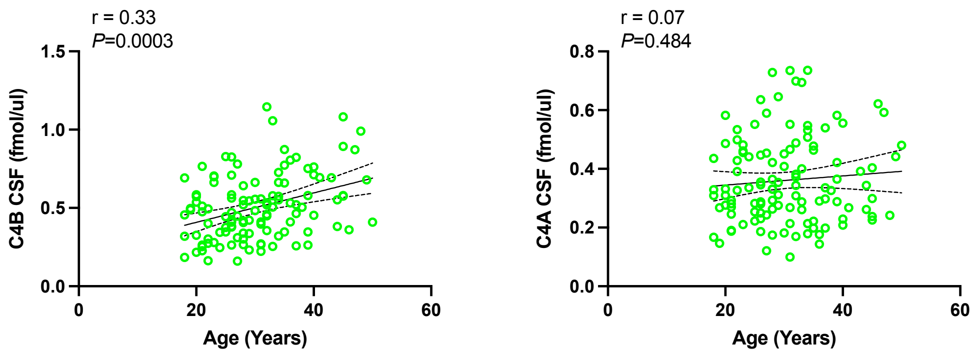

Supplementary Figure 8. Correlation of C4A and C4B cerebrospinal fluid levels with age. C4B was significantly correlated to age in 115 cerebrospinal fluid samples from both cohorts (median age: 29 years; interquartile range 25-35, 83 males and 32 females) (*r*= 0.33; 95% confidence interval [CI]:0.15-0.49; *P*=0.0003, while there was no significant age effect on CSF C4A levels (*r*= 0.07; 95% CI:-0.12-0.25; *P*=0.484). Analyses were performed using Spearman correlation analyses, intercepts with 95% CIs are indicated in the graphs, and p-values are two-sided. Source data is provided as a Source Data file.

Supplementary Figure 9.

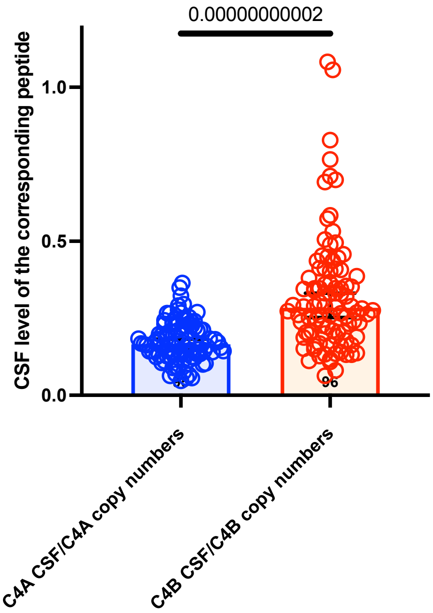

Supplementary Figure 9. Cerebrospinal fluid levels of C4A and C4B per respective copy numbers in the combined discovery and replication cohort. Cerebrospinal fluid (CSF) C4B concentration (fmol/ul) per *C4B* copy numbers (median=0.28, 95% confidence interval CI:0.28-0.36) were significantly higher than corresponding CSF C4A concentration (fmol/ul) per *C4A* copy numbers (median=0.17, CI=0.16-0.10); P=2x10^{11}, n=95. Data was analysed using a Wilcoxon signed-rank test (two-sided p-value). Bar graphs represent medians and error bars represent the 95% confidence interval of each data set. Source data is provided as a Source Data file.

Supplementary Figure 10.

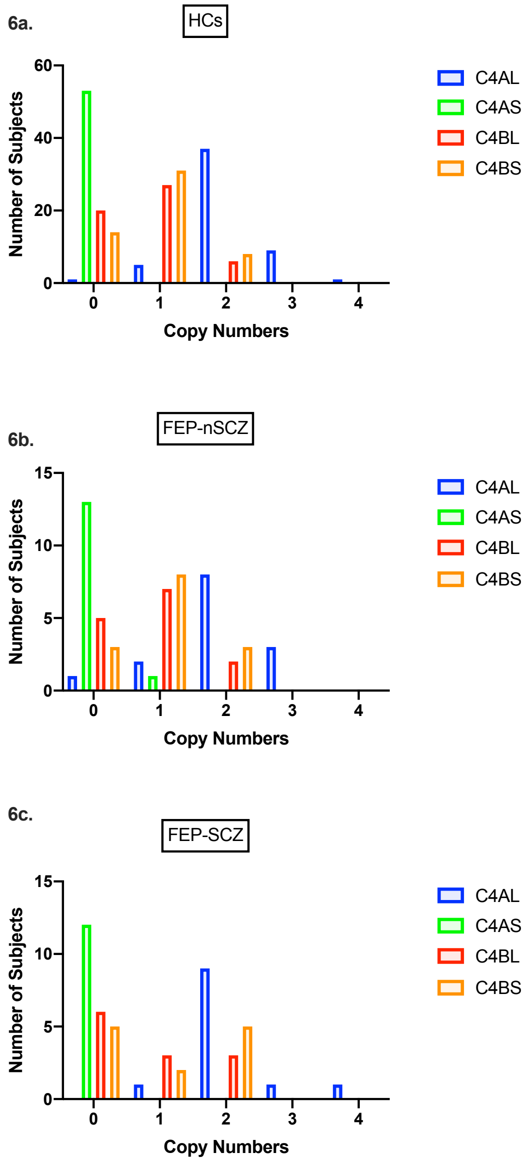

Supplementary Figure 10. Distribution of *C4AL, C4AS, C4BL and C4BS* copy numbers (CNs) in the GRIP cohort. Distribution of *C4AL*, *C4AS*, *C4BL* and *C4BS* in healthy controls (HCs: n=21), patients with first episode psychosis who did not develop schizophrenia (FEP-nSCZ, n=11), and patients with FEP who developed schizophrenia (FEP-SCZ, n=12).

Supplementary Table 1. Demographic and clinical characteristics of the study participants in the discovery sample (KaSP).

	HCs	FEP-nSCZ	FEP-SCZ	P values[1]
	N= 20<br>Mean	N= 15<br>Mean	N= 29<br>Mean	HC vs FEP-nSCZ/<br>HC vs FEP-SCZ/<br>FEP-nSCZ vs FEP-SCZ
Age	27	27	30	0.978/0.311/0.311
Sex (males/females)	12/8	10/5	18/11	0.7372/0.999/0.999
BMI[2]	24[3]	23	23	0.950/0.950/0.950
CSF/Serum Alb ratio[4]	4.8	7.6[5]	6.1[6]	0.538/0.433/0.658
Antipsychotics (Y/N)	0/20	8/7	14/15	0.0003/0.0002/0.999
Tobacco use (Y/N)	2/18	4/11	10/19	0.367/0.738/0.089
Clinical Global Impression (CGI)	NA	4.1	4.6	0.139
Global Assessment of Functioning (GAF)	NA	33	34	0.861
Years of education	14[7]	14	14[8]	0.962/0.950/0.835
AUDIT score	4.2	3.9[9]	4.4[10]	0.993/0.994/0.973
DUDIT score	0.22[11]	2.6[12]	1.0[13]	0.238/0.254/0.584
Type of household-living alone (Y/N[14])	6/10	9/6	7/20	0.289/0.502/0.065
Children in the household (Y/N)	11/5	13/2	24/3	0.394/0.125/0.999
Work/Studies (Y/N)[15]	17/0	9/3	10/9	0.029/0.002/0.274
Subsistence (employed/government assistance)[16]	11/0	9/1	19/6	0.476/0.149/0.645
Number of sick days last year	2	8	2	0.372/0.355/0.942

[1]Healthy controls (HCs), first episode psychosis patients that developed schizophrenia (FEP-SCZ) and first episode psychosis that did not develop schizophrenia (FEP-nSCZ) in the KaSP cohort. Age, BMI, CSF/serum albumin ratio, CGI, GAF, years of education, AUDIT, DUDIT and number of sick days analysed by one way ANOVA test. All other variables were analyzed by Fisher's exact test. Sex, antipsychotics, tobacco use, type of household, children in the household, work/studies and subsistence were analysed by Fisher's exact test. All p values are two sided
[2]BodyMass Index
[3,5,9,10]Data missing for 1 subject
[11]Data missing for 2 subjects
[6,7,13]Data missing for 3 subjects
[8,12]Data missing for 4 subjects
[4]Cerebrospinal fluid (CSF)/Serum albumin ratio
[14]'No' corresponds to living with spouse/with parents/other adults
[15] Yes=Work or studies more than 50%; No=Work or studies less than 50%
[16] Employed=Including unemployment benefits; government assistance= Including sick leave/ subsistance allowance/supported by family or own savings

Supplementary Table 2. Demographic and clinical characteristics of the study participants in the replication cohort (GRIP).

	HCs	FEP-nSCZ	FEP-SCZ	P values[1]
	N=21<br>Mean	N=14<br>Mean	N=17<br>Mean	HC vs FEP-nSCZ/<br>HC vs FEP-SCZ/<br>FEP-SCZ vs FEP-nSCZ
Age	35	33	32	0.518/0.415/0.847
Sex (males/females)	16/5	12/2	15/2	0.676/0.427/0.999
BMI[2]	25	26	27[3]	0.465/0.212/0.497
CSF/Serum Alb ratio[4]	4.9	4.7[5]	5.3	0.784/0.784/0.784
Antipsychotics (Y/N)	0/21	9/5	13/4	0.00004/0.0000007/0.693
Tobacco use (Y/N)	5/16	9/4	6/11	0.014/0.4910/0.139
Relative history of psychiatric illness (Y/N)	NA	8/4	12/2	0.239
Sickness benefit (Y/N)	0/21	4/10	7/10	0.019/0.002/0.707
Social assistance (Y/N)	0/21	2/12	3/14	0.153/0.081/0.999
Unemployment insurance fund (Y/N)	0/21	0/14	1/16	0.999/0.447/0.999
Childhood neurodevelopmental problems (Y/N)[5]	0/21	0/14	0/17	0.999/0.999/0.999
Education (comprehensive school/upper secondary/higher education)	0/9/12	3/4/5	2/8/4	0.087/0.088/0.472

[1]Healthy controls (HCs), first episode psychosis patients that develop schizophrenia (FEP-SCZ) and first episode psychosis that do not develop schizophrenia (FEP-nSCZ) in the GRIP cohort. Age, BMI and CSF/serum albumin ratio analysed by ANOVA test; Sex, antipsychotic, tobacco use, relative history of psychiatric illness, sickness benefit, social assistance, unemployment insurance fund, childhood neurodevelopment problems analysed by Fisher's exact test and education was analysed by Chi square test. All p values are two sided

[2]BodyMass Index

[3,]Data missing for 1 subject

[4]Cerebrospinal fluid (CSF)/Serum albumin ratio

[5]Autism, ADHD, or mental retardation, etc.

Supplementary table 3. Correlations of CSF C4A concentration with change in EMG recordings during habituation to pulse alone trials, and, at the interstimulus interval (ISI) between the prepulse and the pulse at 30, 60, and 120 ms. Data was generated from a total of 44 subjects in the KaSP cohort that had available data (median age: 28 (interquartile range: 25-33; 14 females and 30 males). Exact numbers for each analysis are given in the table. All correlation coefficients were generated using Spearman's correlation analyses. All *P* values are two sided and correction for multiple testing was performed using Benjamini-Hochberg false discovery rate correction.

ISI 30	**r**	**0.21**
	P	0.554
	N	42
ISI 60	r	-0.095
	P	0.675
	N	41
ISI 120	r	0.067
	P	0.675
	N	41
Habituation 1	r	0.25
	P	0.146
	N	43
Habituation 2	r	0.37
	P	0.036
	N	43
Habituation 3	r	0.43
	P	0.036
	N	43
Habituation 4	r	0.29
	P	0.088
	N	44
Habituation 5	r	0.35
	P	0.040
	N	43
Habituation 6	r	0.39
	P	0.036
	N	42
Habituation 7	r	0.24
	P	0.146
	N	41
Habituation 8	r	0.25
	P	0.146
	N	41
Habituation 9	r	0.37
	P	0.036
	N	42
Habituation 10	r	0.39
	P	0.036
	N	42
Habituation 11	r	0.17
	P	0.289
	N	42
Habituation 12	r	0.18
	P	0.254
	N	43
Habituation 13	r	0.32
	P	0.061
	N	43
Habituation 14	r	0.37
	P	0.036
	N	44

Supplementary Table 4. Inclusion list of the light and heavy peptides with their theoretical masses (m) and charge states (z).

Proteins[a]	Peptide Sequence[b]	m/z[c]	z
C4 total	VGDTLNLNLR	557.81	2
	VGDTLNLNL**R**	562.82	2
CO4A	ANSFLGEK	433.22	2
	ANSFLGE**K**	437.23	2
CO4B	ASSFLGEK	419.71	2
	ASSFLGE**K**	423.72	2

[a] C4 total is a peptide representing the total amounts of Complement component C4 (peptide common for isotypes C4A and C4B). CO4A and CO4B are unique peptides for Complement C4A and C4B, respectively.
[b] The bold amino acids are modified.
[c] Mass over charge ratio for the peptides.

Supplementary Table 5: Post-hoc analyses of the non-selected but detectable cytokines on the 10-plex panel against CSF C4A. Analysis was performed using Spearman correlation.

	r	*P*
IL-10	-0.07	0.748
IL-5	0.16	0.45
TNFα	-0.01	0.977
GM-CSF	0.25	0.227
IL-8	0.29	0.149
