# Supplementary material for: Cerebrospinal fluid concentration of complement component 4A is increased in first episode schizophrenia
Source: Nat Commun. 2022 Nov 3;13:6427. doi: 10.1038/s41467-022-33797-6 (PMC9633609; doi:10.1038/s41467-022-33797-6)
Supplement: Supplementary file 3 — Reporting Summary [file 41467_2022_33797_MOESM3_ESM.pdf]

nature portfolio

Corresponding author(s): Carl M. Sellgren

Last updated by author(s): Aug 6, 2022

Reporting Summary

Nature Portfolio wishes to improve the reproducibility of the work that we publish. This form provides structure for consistency and transparency in reporting. For further information on Nature Portfolio policies, see our Editorial Policies and the Editorial Policy Checklist.

Statistics

For all statistical analyses, confirm that the following items are present in the figure legend, table legend, main text, or Methods section.

n/a	Confirmed	
☐	☒	The exact sample size (*n*) for each experimental group/condition, given as a discrete number and unit of measurement
☐	☒	A statement on whether measurements were taken from distinct samples or whether the same sample was measured repeatedly
☐	☒	The statistical test(s) used AND whether they are one- or two-sided<br>*Only common tests should be described solely by name; describe more complex techniques in the Methods section.*
☐	☒	A description of all covariates tested
☐	☒	A description of any assumptions or corrections, such as tests of normality and adjustment for multiple comparisons
☐	☒	A full description of the statistical parameters including central tendency (e.g. means) or other basic estimates (e.g. regression coefficient) AND variation (e.g. standard deviation) or associated estimates of uncertainty (e.g. confidence intervals)
☐	☒	For null hypothesis testing, the test statistic (e.g. *F*, *t*, *r*) with confidence intervals, effect sizes, degrees of freedom and *P* value noted<br>*Give P values as exact values whenever suitable.*
☒	☐	For Bayesian analysis, information on the choice of priors and Markov chain Monte Carlo settings
☒	☐	For hierarchical and complex designs, identification of the appropriate level for tests and full reporting of outcomes
☐	☒	Estimates of effect sizes (e.g. Cohen's *d*, Pearson's *r*), indicating how they were calculated

Our web collection on statistics for biologists contains articles on many of the points above.

Software and code

Policy information about availability of computer code

Data collection	C4 haplotypes were imputed from WGS using Beagle software (version 3.3).
Data analysis	Statistics was performed using R statistics (version 4.2.1), IBM SPSS Statistics (verson 28.0.0.0), or GraphPad Prism (version 9.3.1). Luminex data analysis was performed using MasterPlex QT 2010 (Version 2.0). Targeted mass spectrometry data was analysed using Skyline v4.1.

For manuscripts utilizing custom algorithms or software that are central to the research but not yet described in published literature, software must be made available to editors and reviewers. We strongly encourage code deposition in a community repository (e.g. GitHub). See the Nature Portfolio guidelines for submitting code & software for further information.

Data

Policy information about availability of data

All manuscripts must include a data availability statement. This statement should provide the following information, where applicable:

- Accession codes, unique identifiers, or web links for publicly available datasets
- A description of any restrictions on data availability
- For clinical datasets or third party data, please ensure that the statement adheres to our policy

Source data, necessary to interpret and verify the research in the article, are provided in the Source Data file. In accordance with the institutional regulations and the Swedish law, raw data containing sensitive information that can be used to identify individuals, cannot be shared publicly under the current data protection.

March 2021

Instead, such data can be made available upon request (to the corresponding author) and on a case-by-case basis as allowed by the legislation and ethical permits.

Human research participants

Policy information about studies involving human research participants and Sex and Gender in Research.

Reporting on sex and gender	Information on sex was collected in both cohorts (as defined by the Swedish personal identification number). Sex distribution in patients were not significantly different from the distribution in controls (in either of the cohorts). We observed no effect on sex on CSF C4A or C4B levels. Sex-stratified analyses were not performed as the study was not designed to have sufficient power for such analyses.
Population characteristics	Population characteristics are given in Supplementary Table 1 and 2. Distribution of C4 haplotypes are given in Supplementary Figure 10. For each analyzed sub-cohort we also give demographic info.
Recruitment	Patients were recruited from psychosis units or emergency wards and in- or outpatient facilities at psychiatric clinics in Stockholm or Gothenburg (Sweden). Matched controls were either recruited by advertisement or randomly selected by Statistics Sweden. Given the requirement to give an informed consent, one can expect that some or the more severely sick first-episode psychosis patients declined to participate (given the existing ethical permit, we are however restricted to collect information on patients that decline participation in the study). Further, as substance abuse was an exclusion criteria, patients with a dual diagnosis are per definition not included. Thus, it needs to be considered that severely sick patients with dual diagnosis may display another pattern of CSF C4A and C4B levels than reported here. For healthy controls, at least the subjects recruited by advertisement can in general be considered to be "healthier" than the normal population (selection bias). Nonetheless, e.g., number of sick days last year as well as BMI, tobacco use, and years of education etc. was similar between these controls and patients.
Ethics oversight	The study was approved by the Regional Ethics Committee in Stockholm (Sweden) and the Institutional Review Board of Partners HealthCare (MA, USA). Informed consent was obtained from all included subjects.

Note that full information on the approval of the study protocol must also be provided in the manuscript.

Field-specific reporting

Please select the one below that is the best fit for your research. If you are not sure, read the appropriate sections before making your selection.

☒ Life sciences ☐ Behavioural & social sciences ☐ Ecological, evolutionary & environmental sciences

For a reference copy of the document with all sections, see nature.com/documents/nr-reporting-summary-flat.pdf

Life sciences study design

All studies must disclose on these points even when the disclosure is negative.

Sample size	Available sample size for the initial analysis in the discovery cohort was deemed adequate based on the data presented by Sekar et al (an estimated correlation coefficients of at least 0.5 for C4A (or C4B) CNs versus corresponding RNA expression in 101 subjects, and an odds ratio of 1.4 (P=2x10-5) comparing C4A RNA expression in 35 SCZ subjects and 70 HCs).
Data exclusions	In KaSP, predefined exclusion criteria for patients are ongoing or previous prescription of an antipsychotic for more than 30 days, severe somatic and neurological diseases, current substance abuse (except nicotine use), or autism spectrum disorder. These patients are excluded by clinical examination, medical history, routine laboratory tests, including screening for drugs and MRI scans. For healthy controls, recruited by advertisement and matched on age and sex, eligibility for the study is determined by medical history, routine laboratory tests, clinical examination, and an MRI examination, as evaluated by an experienced neuroradiologist at the MR Centre, Karolinska University Hospital, Solna. The Mini International Neuropsychiatric Interview (MINI), performed by either a resident or a specialist in psychiatry, is used to exclude previous or current psychiatric illness. Further exclusion criteria are former or current use of illegal drugs, first-degree relatives with psychotic illness or bipolar disorder, as well as a neurologic disease and/or severe somatic disease.<br><br>For GRIP cohort, data is collected from patients with schizophrenia spectrum disorders at the psychosis clinic, Sahlgrenska University Hospital, Mölndal, Sweden. The initial diagnostic assessments are performed by treating clinicians at outpatient tertiary-care units, as well as the inpatient units, in Sahlgrenska University Hospital (Sweden). Further clinical information is obtained from interviews at tertiary-care units and investigation units, and when needed supplemented with information from clinical records. To secure diagnoses for research, a series of case conferences are then held (for this study in between October 2017 – March 2018). A minimum of two board-certified psychiatrists participates in these case conferences where a consensus diagnosis is established according to the Diagnostic and Statistical Manual of Mental Disorders, Fifth Edition (DSM-5). The diagnosis is based on structured interviews, medical records (also collected post-baseline), and when needed supplemented with information from the treating psychiatrist. The participating research clinicians are blinded to the results of the CSF analyses. As no matched healthy controls are recruited in GRIP, we used healthy controls from the larger "St. Göran projektet" research project in which GRIP is a sub-study focusing on psychosis. These healthy controls are randomly selected by Statistics Sweden and undergo the same clinical investigations as patients and do not fulfil the criteria for a DSM-IV-TR disorder. From this cohort, we then selected healthy controls to as closely as possible match the patients in GRIP dependent on sex and age.<br><br>All eligible subjects (as described above) with available data were included in the present study. However, one subject from the GRIP did not

display detectable C4A peptide levels but had ddPCR. One subject from the KaSP cohort had indetectable amount of C4B peptide levels. We decided to initially exclude these subjects (one sample from the KaSP cohort and one sample from the GRIP cohort) in the main analyses. However, post-hoc analyses were then also performed including zero values, as well as imputing values with half of the limit of detection, and with similar results (data not shown).

For qPCR analyses, all cultures that passed QC in regard to cell type specification were initially included. However, one culture displayed 100x higher C4A and C4B mRNA expression than all other cultures and was excluded. Further, cultures from one subject did not either show any expression of C4B mRNA, and as we then considered that subject as a non-carrier of the C4B gene these cultures were not further analyzed.

Replication

The main findings in the discovery cohort (CSF data) was replicated in an independent replication cohort. The cellular experiments consisted of three completely independent experiments (with similar results) over multiple independent cultures from four patients to account for variability between subjects, within subjects (derivations), and in regard to cytokine stimulations.

Randomization

We used an observational design (i.e., no randomization). According to the pre-defined analytical plan, a significant increase in C4A (or C4B) concentration in the discovery cohort (P<0.05, two-sided p-value) was to be followed-up in an independent cohort then again measuring C4A, as well as C4B, with significance set at P<0.05 (two-sided p-value). Potential confounders were to be tested on group levels (in each cohort). If this indicated any evidence of between group differences (on a liberal p-value threshold of 0.1), indicated variables were to be studied in relation to CSF C4A or C4B concentration. If this revealed an association with P<0.05, we were to perform an adjusted analysis. The exception to this rule was antipsychotics, here we before analyses decided to 1) compare levels between medicated and non-medicated patients, and 2) even if we observed no indication of altered levels in medicated vs. unmedicated we were to perform stratified/adjusted analyses.

Blinding

All researchers performing mass spectrometry and Luminex analyses were blinded to the identity of the samples. Clinical information (baseline and follow-up), including diagnosis, were collected prior to collection of biological data, i.e., the particpating reserach clinicans were blinded to the results of the CSF analyses, genotypes, etc.
The identity of individual cell lines used for cytokine stimulations were blinded to the person performing the stimulation and the qPCR experiments.

Reporting for specific materials, systems and methods

We require information from authors about some types of materials, experimental systems and methods used in many studies. Here, indicate whether each material, system or method listed is relevant to your study. If you are not sure if a list item applies to your research, read the appropriate section before selecting a response.

Materials & experimental systems

n/a	Involved in the study	
☐	☒	Antibodies
☐	☒	Eukaryotic cell lines
☒	☐	Palaeontology and archaeology
☒	☐	Animals and other organisms
☒	☐	Clinical data
☒	☐	Dual use research of concern

Methods

n/a	Involved in the study	
☒	☐	ChIP-seq
☒	☐	Flow cytometry
☒	☐	MRI-based neuroimaging

Antibodies

Antibodies used

Primary:
MAP-2 (Abcam, Cat# ab5392)
Beta III Tubulin (Promega, Cat# G7121)
OCT 4 (Cell signaling 2840) Rabbit 1:400
TRA 1-60 (Abcam Ab16288) Mouse 1:500

Secondary:
Goat anti-chicken (ThermoFisher Scientific, Cat# A32933)
Goat anti-mouse (Abcam, Cat# ab150113).
Goat anti-Rabbit (Abcam, Catalog # A32732)

Validation

MAP-2 (Abcam, Cat# ab5392): The Abpromise guarantee covers the use of ab5392 for ICC applications. Sensitivity and specificity was also tested in-house for this cell type.

Beta III Tubulin (Promega, Cat# G7121): The ab is recommended for ICC by Promega, and sensitivity/specificity was also tested in-house for this cell type.

OCT4 (Cell signalling, Cat# 2840): The ab is recommended for ICC by Cell signalling Technology at a concentration between 1:200 and 1:500, and sensitivity/specificity was also tested in-house for this cell type and has been routinely used to validate iPSC lines in the MGH Neurobank.

Tra-1-60 (Abcam, Cat# Ab16288): The ab is recommended for ICC by Abcam (covered for this application by the Abpromise guarantee) at a concentration of 1:500, and sensitivity/specificity was also tested in-house for this cell type and has been routinely used to validate iPSC lines in the MGH Neurobank.

Eukaryotic cell lines

Policy information about cell lines and Sex and Gender in Research

Cell line source(s)	The human iPSC lines used were obtained from MGH Neurobank under an MTA.
Authentication	All the iPSC lines were validated by positive staining for octamer-binding transcription factor 4 (POU domain, class 5, transcription factor 1) and TRA-1–60, performed by the MGH Neurobank.
Mycoplasma contamination	All lines tested negative for mycoplasma contamination.
Commonly misidentified lines (See ICLAC register)	No commonly misidentified lines were used.
